# Instrumental Activities of Daily Living Tools in Very-Low Vision: Ready for Use in Trials?

**DOI:** 10.3390/pharmaceutics14112435

**Published:** 2022-11-10

**Authors:** Jan Henrik Terheyden, David J. Fink, Susanne G. Pondorfer, Frank G. Holz, Robert P. Finger

**Affiliations:** Department of Ophthalmology, University Hospital Bonn, 53127 Bonn, Germany

**Keywords:** activities of daily living, low vision, vision restoration, clinical trial endpoints

## Abstract

Traditional endpoints assessing visual function are limited by their responsiveness to interventions restoring or maintaining vision. An alternative concept is assessing instrumental activities of daily living (IADL). Herein, we review all available vision-specific IADL instruments relevant for vision restoration trials and report data for the most promising instrument. Six relevant instruments exist: The Low Vision Functional Status Evaluation (LVFSE), Timed IADL (TIADL), Melbourne Low-Vision Activities of Daily Living Index (MLVAI), Assessment of Disability Related to Vision (ADREV), Functional Low-Vision Observer Rated Assessment (FLORA), and Very Low Vision IADL (IADL-VLV). Both internal consistency and test-retest data were available for the LVFSE, MLVAI, and IADL-VLV. In a sample from a low-vision clinic (*n* = 51; age 57 ± 16 years), we report additional validation data on the IVI-VLV including test–retest reliability (intraclass correlation coefficient 0.981 [0.961; 0.991]). The LVSFE was noticeably less reliable than the MLVAI and the IADL-VLV. Content and construct validity data were available for the LVFSE, TIADL, MLVAI, ADREV, and IADL-VLV, but only the MLVAI and IADL-VLV were developed for an ultra-low vision context. Ceiling effects were present across instruments. Thus, of all appropriate IADL instruments related to vision, the IADL-VLV and MLVAI best meet existing requirements for use in vision restoration trials, e.g., in gene therapies or visual prostheses in inherited retinal diseases, but require further validation.

## 1. Introduction

About 80% of sensory information processed by human brains is acquired by the visual system [1]. Visual impairment and blindness thus affect people in numerous activities during their everyday lives. Retinal diseases are among the most common causes of irreversible visual disability worldwide and account for more than 25% of irreversible visual impairments and more than 15% of cases of irreversible blindness globally [2]. Inherited retinal diseases are particularly devastating since their onset is typically at a younger age than that of acquired retinal diseases, and many of those diseases still have a poor prognosis and currently lack causal therapeutic options [3]. However, with the ongoing technological advances, innovative therapeutic approaches are increasing including gene- and cell-based therapies as well as visual prostheses. These interventions primarily target patients with very- or ultra-low vision. Traditional ophthalmic endpoints including conventional functional tests, such as visual acuity, do not capture overall functional abilities well in individuals with little residual vision and are unresponsive to only small changes in functional performance achieved with many novel sight-restoring interventions. At the same time, regulatory authorities prefer endpoints that reflect individuals’ functional difficulties over purely structural metrics. Patient-reported outcome and performance-based tests may bridge this gap. For example, Activities of Daily Living (ADL) related to vision reflect global visual function in very- and ultra-low vision patients better than do conventional functional assessments [4,5,6,7]. Thus, we provide an overview of existing vision-specific ADL instruments and their status of validation in the context of vision restoration trial endpoints.

## 2. Materials and Methods

### 2.1. Activities of Daily Living

The first performance-based tests (PBTs) were used to measure independence and functional capabilities in older people when in the 1960s, Sidney Katz and colleagues validated a standardized set of tasks designed to measure the newly defined concept of ADL [8]. These cover basic skills necessary to maintain self-care, self-feeding, or mobility. Only a few years later, the concept of ADL was extended to more complex activities such as handling personal finances, meal preparation, shopping, taking medication, or doing housework, named instrumental activities of daily living (IADLs) [9]. Since then, a variety of IADL tools have been developed, including instruments specifically assessing IADL related to vision.

### 2.2. IADL Instruments in Low Vision

Vision researchers extended the concept of IADL over time, adding novel domains such as picture recognition and reading tests to the instruments. As a consequence, vision-specific ADL and IADL instruments are noticeably heterogeneous [4]. This contributes to reliability and validity issues seen in many of the longer-existing instruments, which has also been highlighted by a panel of experts from academia, industry, and the United States Food and Drug Administration (FDA) [10].

### 2.3. Search Strategy

For this reason, we have focused this review on instruments that meet a more traditional definition of IADL, understand IADL assessments as multidimensional, and have undergone at least the first steps of validation. For this purpose, we have searched the medical literature for IADL tools used in the context of ophthalmology, using PubMed. The search terms were the Medical Subject Headings “Activities of Daily Living” and “Low Vision”. We included only publications available in English. Non-vision-specific instruments as well as instruments assessing one domain only (e.g., reading performance or mobility only) were excluded.

## 3. Results

We describe the identified instruments below; further information on the validation status can be found in Table 1.

### 3.1. Low Vision Functional State Evaluation (1999)

The Low Vision Functional Status Evaluation (LVFSE) was developed to measure the effectiveness of vision rehabilitation programs. It includes 27 items that cover both IADL tasks and reading tasks [11]. All items were selected to specifically target vision-related disabilities and cover areas such as meal preparation, shopping, taking medicine, communication, handling finances, and home maintenance. Although most participants of the validation study underwent low vision rehabilitation, the cohort also included participants with mild visual limitations.

### 3.2. Timed Instrumental Activities of Daily Living (2001)

Owsley et al. developed the timed IADL tool (TIADL) to assess everyday performance in elderly people related to vision and memory with the goal of identifying items suitable for use in low vision trials [12]. The final version of the instrument consists of 5 timed tasks that are relevant ADLs. Accuracy is estimated based on the summary of individual scores. Time penalties are added when the performance of individual items is successful but inaccurate [12,13]. The authors especially used the TIADL to examine the relationship between vision and cognition.

### 3.3. Melbourne Low-Vision ADL-Index (2001)

The Melbourne Low-Vision ADL index (MLVAI) was specifically designed to assess visual performance in a low-vision population for visual rehabilitation [14]. The MLVAI contains two parts: Eighteen observed IADL items from areas such as meal preparation, shopping, taking medicine, communication, handling finances, and home maintenance, as well as 9 self-reported items concerning self-care ADL. All items are rated according to observed or self-perceived speed, accuracy, and level of independence on a five-step Likert scale [14,15,16].

### 3.4. Assessment of Disability Related to Vision (2009)

The Assessment of Disability Related to Vision (ADREV) contains 9 items and was originally developed based on existing work with two other instruments, the Assessment of Function Related to Vision (AFREV) and the Task Performance Test (TPT) [17]. It was developed for clinical research and ophthalmic practice settings. Each item is rated using seven difficulty levels. Its performance has been evaluated in different populations with age-related macular degeneration, glaucoma, and diabetic retinopathy [18,19].

### 3.5. Functional Low-Vision Observer Rated Assessment (2014)

The Functional Low-Vision Observer Rated Assessment (FLORA) was developed in the context of a trial for a retinal prosthesis device (Argus II) and is thus for people with ultra-low vision [20]. It has three parts, including a PBM, a self-reported part, and a qualitative assessment. The FLORA has only been validated in a small group of 26 individuals. Performance is assessed by the evaluator on a 4 step Likert scale but without timing.

### 3.6. Very Low Vision Instrumental Activities of Daily Living (2014)

The IADL-VLV was specifically designed to assess performance in IADLs in individuals with very-low vision and potentially serve as a vision restoration trial endpoint, within the Bionic Vision Australia Retinal Prosthesis Project [21]. Its development included a noticeable proportion of qualitative steps such as a Delphi consensus procedure. The IADL-VLV assesses performance based on accuracy and time and has not been widely validated to date. For example, no repeatability data have been published for the IADL-VLV, which is why we assessed its psychometric performance including its test–retest repeatability in a cohort of patients (see below).

**Table 1:** Overview of IADL instruments.

Instrument	Validation Cohorts	Domains	Outcome Type	Reliability Data	Validity Data ^#^
LVFSE	- 155 individuals	14 (initially 27) items on reading, text search, object search, object identification, fine motor skills	- Time - Working distance - Accuracy - Observed task difficulty - Subjective task difficulty	Internal consistency	Content validity:
	[11]		*(Multiple separate scores)*	- Average α = 0.84	
				Test–retest	
				- Average r = 0.52	- Expert opinion - Literature review
				Interrater	Construct validity:
				- Average r = 0.73	- r = 0.6 (VA) Explained variability by Visual clinical state in multiple regression analysis
TIADL	- 342 older adults with cataracts - 173 older adults - 97 older adults	17 (reduced: 5) items on reading, text search, object search, fine motor skills	- Time (including accuracy as time penalty)	Test–retest	Content validity:
	[12] [13] [22]		*(Multiple scores; single score for reduced version)*	- r = 0.85	- Literature review
					Construct validity:
					- r = 0.09 to 0.36 (with VA), 0.09 to 0.39 (with CS), 0.04 to 0.3 (with VF) - Responsiveness (training): - *p* = 0.049
MLVAI	- 122 patients with low vision - 97 individuals with vision impairment - 22 individuals with age-related macular degeneration	16 (initially 18) IADL items on reading, text search, human interaction, fine motor skills, sign reading + 9 self-reported ADL items	- Accuracy, time, subjective independence	Internal consistency	Content validity:
	[14] [15] [16]		*(Single score)*	- α = 0.94–0.96 - PR = 0.96, PSI = 4.70	- Literature review
				Test–retest	Construct validity:
				- ICC = 0.88–0.95	- r = −0.68 (with VA/VF)
				Interrater	Responsiveness (rehabilitation)
				- r ≥ 0.9	- effect size 0.78
				- ICC = 0.97 (15 raters on 2 study participants)	
ADREV	- 112 patients with AMD - 91 patients with DR - 194 patients with glaucoma	9 items on reading, human interaction sign reading, motion recognition, object search, navigating, fine motor skills, object matching	- Accuracy	Internal consistency	Content validity ^§^:
	[4] [17] [18]		*(Single score)*	- PR = 0.86, PSI = 2.5 in AMD	- Literature review - Expert opinion
					Construct validity
					- r = 0.52 to 0.82 (with VA), 0.70 (with CS), 0.30 to 0.58 (with VF) in AMD - r = 0.62 (with NEI VFQ) in AMD - r = 0.57 to 0.78 (with VA), 0.61 (with CS), 0.56 to 0.57 (with VF) in DR - r = 0.50 (with NEI VFQ) in DR - r = 0.79 (with VA), 0.80 (with CS), 0.42 (with VF) in glaucoma - r = 0.55 (with NEI VFQ) in glaucoma
FLORA	- 26 individuals with ultra-low vision [20]	35 items on object search, navigating, object identification, object matching (tested at participants’ home)	- Accuracy	Not available	Content validity:
			*Additionally:*		
			- Narrative case report - Self-reported difficulty		- Expert consensus - Literature review
			*(Multiple separate scores)*		Responsiveness (retinal prosthesis):
					- significant improvement in 69% of tasks
IADL-VLV	- 40 individuals with very-low vision - 51 individuals from a low-vision clinic	11 (initially 29/23) items on object search, sign reading, fine motor skills, object matching, human interaction	- Accuracy, including number of attempts - Time	Internal consistency	Content validity:
	[21]		*(Separate scores)*	- PR 0.81–0.93, PSI 2.05–3.8	
				Test–retest	- Literature review - Expert input - Patient consensus (Delphi method)
				- ICC = 0.98 [accuracy], −0.01–0.95 [time]	Construct validity:
					- r = 0.62 to 0.81 (VA), 0.04 to 0.95 (VF) [accuracy]; r = 0.46 to 0.73 (VA), 0.18 to 0.50 (VF) [time] - r = 0.62 to 0.81 (accuracy), 0.46 to 0.73 (time) [VA] - r = 0.04 to 0.95 (accuracy), 0.18 to 0.50 (time) [VF] - In multivariate analyses only association with VA and VF

ADL—activities of daily living; ADREV—Assessment of Disability Related to Vision; AMD—age-related macular degeneration; CS—contrast sensitivity; DR—diabetic retinopathy; FLORA—Functional Low-Vision Observer Rated Assessment; IADL-VLV—Very Low Vision Instrumental Activities of Daily Living; ICC—intraclass correlation coefficient; LVFSE—Low Vision Functional Status Examination; MLVAI—Melbourne Low-Vision Activities of Daily Living Index; NEI VFQ—National Eye Institute Visual Function Questionnaire; PR—person reliability; PSI—person separation index; TIADL—Timed Instrumental Activities of Daily Living; VA—visual acuity; VF—visual field. ^#^ For comparability across instruments, provided R^2^ values obtained from regression models were transformed to correlation coefficients. ^§^ The authors consider the ADREV the third generation of the same performance-based measure. The first and second generations of this instrument (Task Performance Test, TPT; Assessment of Function Related to Vision, AFREV) were thus not specifically included in this table but referenced when applicable.

### 3.7. IADL-VLV Study Methods

Participants were recruited from a low-vision clinic at the outpatient department of the University Hospital Bonn, Germany and provided written, informed consent prior to participating. The institution’s ethics committee approved the study before it was started, and all procedures were in accordance with the tenets of the Declaration of Helsinki. The inclusion criterion was at least moderate visual impairment based on reduced visual acuity or visual fields. Exclusion criteria were cognitive impairment, a recent (within 3 months) change in visual impairment or change in visual impairment between repeated assessments, and any ocular surgery within three months prior to study start. All participants were tested with the IADL-VLV, following a method described previously [21]. The assessments were performed by one staff member specifically trained for this purpose. A Rasch model was fit to the dataset, the psychometric performance was assessed, and the item set of the IADL-VLV was subsequently reduced based on the item fit statistics. Intraclass correlation coefficients between the test and retest assessments were calculated based on person measures from the final Rasch model. As an additional outcome measure beyond the existing scale, we assessed the repeatability of item timing based on ICCs. Statistical analyses were conducted with Winsteps (version 3.92.1; Portland, OR, USA) and IBM SPSS Statistics (version 27; Armonk, NY, USA). *p*-values < 0.05 were considered statistically significant.

### 3.8. IADL-VLV Study Results

We recruited 51 individuals (women 37.3%; men 62.7%) with a mean better eye visual acuity of 1.04 ± 0.69 LogMar. The diagnoses of participants included retinitis pigmentosa (27.5%), age-related macular degeneration (19.6%), cone dystrophies (13.7%), Stargardt disease (15.6%), previous retinal detachments (5.9%), and other congenital or hereditary conditions impairing vision (17.6%). Test–retest data were available for 31 participants. Several Rasch model requirements were violated with the original version of the IADL-VLV, and we thus modified the instrument based on our data (Table 2). First, we collapsed two of the four categories (task completed inaccurately in 1–2 attempts and task completed inaccurately in more than 2 attempts) due to disordered thresholds in two items. This led to all thresholds being ordered, but several items showed misfit. We first removed six items with a distorting level of misfit (mean-square values > 2.0). After omitting a misfitting response to one item, twelve further misfitting items (outside the infit mean-square value corridor 0.5–1.5 [21]) were dropped from the scale. The resulting 11-item version of the IADL-VLV had no misfitting items and an internal consistency within recommended limits but showed evidence of multidimensionality (Table 2). However, splitting the scale into two separate subscales led to an instability in the scale, and we thus proceeded with one global scale.

The baseline scores of the 11-item version of the IADL-VLV were significantly associated with the visual acuity in the better-seeing eye, with a correlation coefficient (Pearson) of −0.673. The average score on the 11-item IADL-VLV was 2.9 ± 2.2, the mean absolute difference between test and retest assessments was 0.25 ± 0.45, and the revised instrument had an ICC of 0.981 [95% confidence interval 0.961; 0.991], but ceiling effects were observed, which is why we subsequently investigated timing of the individual items as an additional outcome measure (Table 3).

Of the eleven items included in the revised version of the IADL-VLV, nine items showed positive intraclass correlations of timing data that were significantly different from 0 (Table 3). Moreover, the association between these two items as well as three additional items (“Coffee mug”, “Bowl”, “Hankies colored”) were not significantly correlated with the better eye visual acuity (other items: Pearson r coefficients 0.30 to 0.48, *p* ≤ 0.038). In order to improve the 11-item instrument, we considered dropping the items “Dinner plate” and “Dinner spoon” from the Rasch model, but this resulted in a decline of the person separation index to 1.95. For this reason, we consider the 11-item IADL-VLV the revised version of the instrument and recommend it for use in future studies instead of the longer version.

## 4. Discussion

A number of vision-related IADL instruments exist, none of which has been validated to an extent required for clinical trial endpoints. Providing additional validation steps and assessing the psychometric performance of one of the better-suited IADL instruments available, we found ceiling effects for the majority of items as well as several psychometric issues with the IADL-VLV. Removing items, restructuring its rating scales, and employing additional outcome ratings such as time improved its psychometric performance to acceptable levels. Both the literature review and the psychometric evaluation of the IADL-VLV highlight that the measurement of vision-related IADLs comes with considerable challenges.

First and foremost, in the initial design, including the selection of appropriate items in IADL tools is crucial. Only a proportion of the mentioned instruments (LVFSE, ADREV, FLORA, IVI-VLV) reported including structured feedback from experts and/or patients in the selection of tasks, while the other instruments were constructed based on literature review only, which may lead to such instruments not being patient-relevant. The IADL-VLV was the only instrument which qualitatively implemented patient feedback during its development phase, using Delphi methodology. The level of reliability testing varied noticeably between instruments. Both internal consistency and test–retest reliability were reported only for the LVFSE, MLVAI, and IADL-VLV. While the data reported for the MLVAI and the IADL-VLV suggested that these instruments can be considered reliable, the test–retest correlation of the LVFSE was only 0.5, and this instrument cannot be recommended for further use on this basis. Not all identified IADL instruments were specifically developed for use in a very- or ultra-low vision population (LVFSE, MLVAI, FLORA, IADL-VLV), which may limit the content validity of the respective tests. Four of the instruments were reported to be construct valid based on associations with visual function tests or patient-reported outcome measures, i.e., the LVFSE, TIADL, MLVAI, ADREV, and IADL-VLV. Of these instruments, first responsiveness data have been published for the TIADL and the MLVAI, as well as for the FLORA, supporting the instruments’ responsiveness to an intervention.

Based on our evaluation of the scientific quality criteria of IADL instruments in the context of clinical trials in very- or ultra-low vision, only the MLVAI and the IADL-VLV have passed important validation steps so far and were constructed for use in a low-vision population, making these instruments particularly promising for further evaluation and responsiveness testing in a clinical trial context. While the validation cohorts of the MLVAI were larger (overall *n* = 241; IADL-VLV: overall *n* = 91), the IADL-VLV has been designed based on more qualitative work and comes with two types of outcome assessments (accuracy and time), whereas the MLVAI generates only a single outcome measure (accuracy). This makes the IADL-VLV match the recommendations for the development and properties of tools assessing ADL-based vision-related performance testing better than does the MLVAI since these recommendations include speed and accuracy in the scoring strategy [23].

The overall range of activity categories assessed by the different instruments is relatively wide. Nevertheless, most tasks fall into the categories of “searching tasks”, “fine motor tasks”, “reading and identification tasks”, or “navigation tasks”. All listed instruments include the searching, fine motor, and reading task categories while the navigation category is only included in the ADREV and the FLORA. Even though this may increase an instrument’s validity, navigation-specific tools that are conducted using obstacle courses under prespecified laboratory conditions seem more appropriate to quantify individuals’ difficulties navigating under a clinical trial setting than do the tests included in the ADREV, which includes one navigation item only, or the FLORA, which is home-based. Tasks related to reading words or texts are included in the TIADL, LVFSE, MLVAI, and ADREV, which may not be feasible for many individuals with very- or ultra-low vision, depending on the individual visual function. We thus recommend multidimensional tools for future vision restoration trials. The reduced, 11-item version of the IADL-VLV meets these criteria and includes tasks from the searching, fine motor, and reading/identification domains. Against this background, we recommend the IADL-VLV with 11 items for further validation in other low-vision cohorts including testing of responsiveness to effective therapeutic interventions.

The first IADL instruments described in this article were developed more than 20 years ago, but their use in interventional trials is still relatively sparse, which is partly due to only a handful of sight-restoring or similar interventions having been tested in clinical trials. A strong argument for implementing more IADL endpoints is their content validity (direct relation to patients’ daily lives). Despite this, we have outlined that only a minority of the existing instruments meet other basic psychometric quality criteria and that the remaining instruments have not been extensively studied in interventional settings thus far, which comes with risks for the sponsors of large-scale, costly trials selecting their endpoints. This is also reflected by the recommendations of the Harmonization of Outcomes and Vision Endpoints in Vision Restoration Trials (HOVER) taskforce [23]. To improve this evidence and add IADL instruments to the toolbox, clinical research with approved therapies and in the field of visual rehabilitation is required.

Patient-reported outcome measures (PROMs) designed specifically to be used in a low-vision population are an alternative to performance-based tests (PBTs). The Impact of the Vision Impairment–Very Low Vision (IVI-VLV) questionnaire and the Veterans Affairs Low-Vision Visual Functioning Questionnaire (VA LV-VFQ) are two examples of instruments that have already passed initial validation steps [24,25]. When compared to PBTs, PROMs have the advantage of being self-administered and therefore require less resources by clinical site staff. However, adequately designed PBTs may be less variable and have better repeatability over PROMs since they have been shown to be less affected by, for example, neuro-psychiatric comorbidities [6,26,27,28]. In addition, the development of compensatory mechanisms for ADL tasks is associated with the onset of visual impairment. This suggests that PBTs might be particularly useful in rapidly progressive diseases for which such strategies have not yet been developed [29]. Depending on the intended use, both PROMs and PBTs may have advantages over one or the other, or may yield complementary information. The choice of endpoints should as always be informed by the research question posed.

Our work has several strengths and limitations. We performed a focused literature review concentrating on PBTs of vision-related IADLs and performed additional evaluation of one of the best-suited PBTs. It is unlikely that we missed relevant PBTs or any studies evaluating these, as the field is small and we have worked with these PBTs for many years. We assessed the repeatability of the IADL-VLV in a relatively small and heterogeneous population with different levels of visual impairment. A large proportion of the participants (49%) met the very low-vision criterion in both eyes, which makes the sample comparable to those studies in the reviewed literature. Lastly, we were not able to provide responsiveness data for the IADL-VLV to an intervention, which we consider future work.

## 5. Conclusions

In conclusion, only few multidimensional PBTs under ongoing validation are available for use in future vision restoration trials. The validation status of the IADL-VLV and the MLVAI is most advanced in the field of low-vision PBTs assessing IADLs and show promising results, but longitudinal studies are currently lacking and are required for the assessment of, for example, responsiveness to change over time or minimally important difference, both of which are required for clinical trial endpoints.

## Figures and Tables

**Table 2:** Rasch model characteristics of the Very Low Vision Instrumental Activities of Daily Living.

Parameters	Rasch Model	IADL-VLV-29	IADL-VLV-11
Disordered thresholds, n	0	**2**	0
Misfitting items, n	0	**12**	0
PSI	>2.0 (1.5)	**1.29**	2.05
PR	>0.8 (0.6)	**0.63**	0.81
Difference in person and item mean	<1	**2.98**	**2.46**
PCA, 1st contrast eigenvalue	<2	**11.5**	**5.69**

PCA—principal component analysis; PR—person reliability; PSI—person separation index. Values displayed in bold violate the Rasch model requirements shown in the left column.

**Table 3:** Very Low Vision Instrumental Activities of Daily Living baseline item characteristics and intraclass correlation in a repeated test.

Outcome		Success		Time		
Task	Rasch Model	Failed (%)	Cohen’s κ [95% CI]	Mean ± SD	Δ Mean ± SD	ICC [95% CI]
Placemat	✗	3 (6.1)	**1.0 [1.0; 1.0]**	5 ± 14	4 ± 4	0.131 [−0.722; 0.561]
**Dinner plate**	✓ *	3 (6.1)	**1.0 [1.0; 1.0]**	5 ± 17	3 ± 16	0.001 [−0.979; 0.496]
**Coffee mug**	✓	3 (6.1)	**1.0 [1.0; 1.0]**	3 ± 4	1 ± 1	**0.791 [0.587; 0.895]**
**Bowl**	✓	4 (7.8)	**0.654 [0.027; 1.0]**	3 ± 3	1 ± 3	**0.683 [0.366; 0.842]**
Dinner fork	✗	4 (7.8)	**1.0 [1.0; 1.0]**	4 ± 9	2 ± 10	0.002 [−0.976; 0.496]
Dinner knife	✗	4 (7.8)	**0.654 [0.027; 1.0]**	4 ± 9	2 ± 10	−0.006 [−1.0; 0.498]
**Dinner spoon**	✓ *	3 (6.1)	**1.0 [1.0; 1.0]**	4 ± 8	2 ± 10	−0.012 [−1.0; 0.489]
**Clock 1**	✓	3 (6.1)	**1.0 [1.0; 1.0]**	6 ± 10	2 ± 5	**0.870 [0.740; 0.935]**
Clock 2	✗	3 (6.1)	**1.0 [1.0; 1.0]**	5 ± 6	2 ± 3	**0.933 [0.866; 0.966]**
Clock 3	✗	3 (6.1)	**1.0 [1.0; 1.0]**	5 ± 6	2 ± 3	**0.928 [0.856; 0.964]**
Clock 4	✗	3 (6.1)	**1.0 [1.0; 1.0]**	5 ± 6	2 ± 4	**0.889 [0.779; 0.945]**
Sign “right arrow”	✗	3 (6.1)	**1.0 [1.0; 1.0]**	10 ± 31	1 ± 2	0.371 [−0.259; 0.686]
Sign “first aid/cross”	✗	3 (6.1)	**1.0 [1.0; 1.0]**	5 ± 12	2 ± 11	**0.620 [0.240; 0.810]**
Sign “steps”	✗	3 (6.1)	**1.0 [1.0; 1.0]**	4 ± 7	0 ± 0	**0.999 [0.998; 0.999]**
Sign “down arrow”	✗	3 (6.1)	**1.0 [1.0; 1.0]**	4 ± 9	1 ± 2	**0.961 [0.923; 0.981]**
Sign “man”	✗	3 (6.1)	**1.0 [1.0; 1.0]**	5 ± 11	0 ± 1	**0.996 [0.991; 0.998]**
Sign “coffee/cup”	✗	3 (6.1)	**1.0 [1.0; 1.0]**	5 ± 10	1 ± 1	**0.989 [0.979; 0.995]**
**Signature small**	✓	3 (6.1)	**1.0 [1.0; 1.0]**	3 ± 1	0 ± 1	**0.887 [0.774; 0.944]**
**Signature medium**	✓	3 (6.1)	**0.654 [0.027; 1.0]**	3 ± 2	1 ± 1	**0.935 [0.869; 0.967]**
Signature large	✗	1 (2.0)	Not determinable ^#^	5 ± 11	2 ± 8	**0.865 [0.736; 0.931]**
Singlets 2 × 3	✗	1 (2.0)	Not determinable ^#^	18 ± 26	3 ± 7	**0.984 [0.970; 0.992]**
Singlets 3 × 2	✗	2 (3.9)	**1.0 [1.0; 1.0]**	23 ± 14	6 ± 9	**0.881 [0.765; 0.940]**
Socks	✗	1 (2.0)	Not determinable ^#^	26 ± 33	8 ± 14	**0.954 [0.910; 0.977]**
Hankies light/dark	✗	2 (3.9)	**1.0 [1.0; 1.0]**	28 ± 28	5 ± 6	**0.983 [0.966; 0.991]**
Hankies light/dark pattern	✗	2 (3.9)	**1.0 [1.0; 1.0]**	35 ± 34	9 ± 15	**0.944 [0.889; 0.972]**
**Hankies colored**	✓	4 (7.8)	**1.0 [1.0; 1.0]**	57 ± 58	12 ± 22	**0.947 [0.895; 0.974]**
**Gesture “quiet”**	✓	10 (19.6)	**0.748 [0.478; 1.0]**	5 ± 5	1 ± 2	**0.803 [0.568; 0.910]**
**Gesture “beckoning”**	✓	5 (9.8)	**1.0 [1.0; 1.0]**	4 ± 5	1 ± 2	**0.895 [0.785; 0.949]**
**Gesture “stop”**	✓	6 (11.8)	**0.785 [0.379; 1.0]**	4 ± 4	1 ± 2	**0.931 [0.857; 0.967]**

CI—confidence interval; ✓—included in model; ✗—excluded from model. Repeatability data printed in bold were significantly different from 0. * Omitted in a 9-item version; ^#^ Coefficient not determinable when test or retest values are constant. Tasks displayed in bold were included in the 11-item Rasch model. Cohen’s κ values and ICCs displayed in bold were significantly different from 0.

## Data Availability

The original data presented in this study are available on request from the corresponding author. The data are not publicly available due to privacy restrictions.
